# Development of a laboratory-based nomogram for predicting clinical outcomes in patients with severe COVID-19 undergoing glucocorticoid therapy

**DOI:** 10.3389/fmed.2025.1635545

**Published:** 2025-12-05

**Authors:** Langqing Xu, Chunyang Hou, Jing Jie, Zhifang Jia, Lei Song, Dan Li

**Affiliations:** 1 Department of Respiratory Medicine, Center for Infectious Diseases and Pathogen Biology, State Key Laboratory for Diagnosis and Treatment of Severe Zoonotic Infectious Diseases, The First Hospital of Jilin University, Changchun, China; 2 Department of Urology, The First Hospital of Jilin University, Changchun, Jilin, China; 3 Division of Clinical Epidemiology, The First Hospital of Jilin University, Changchun, China

**Keywords:** severe COVID-19, glucocorticoid therapy, nomogram model, cytokine storm, predictive biomarkers, ferritin, Interleukin-10

## Abstract

**Background:**

While glucocorticoids remain cornerstone therapy for severe COVID-19, substantial heterogeneity persists in clinical outcomes. This single-center retrospective study sought to establish a predictive model integrating inflammatory biomarkers to guide risk stratification and personalized management.

**Methods:**

We analysed 151 adults with WHO-defined severe COVID-19 receiving glucocorticoid therapy (December 2022–August 2023). Treatment non-response was defined as mortality during hospitalization, mechanical ventilation escalation, or persistent organ dysfunction. LASSO and logistic regression analyses identified predictors, with optimal biomarker thresholds determined using ROC curves. A nomogram was constructed and validated via split-sample testing (7:3 ratio) and 10-fold cross-validation.

**Results:**

Ferritin >970.7 ng/mL and IL-10 > 4.79 pg./mL predicted glucocorticoid resistance (AUC: training set 0.779, test set 0.780). The nomogram incorporated diabetes, ferritin, and IL-10, demonstrating robust calibration (Hosmer-Lemeshow *p* = 0.84; Brier score = 0.182) and discrimination (sensitivity = 71.4%, specificity = 70.0%). Diabetic patients exhibited heightened inflammatory responses and poorer outcomes, exacerbated by glucocorticoid-induced hyperglycaemia.

**Conclusion:**

This nomogram shows promising predictive performance and provides a potentially implementable framework for risk stratification and personalized management, which warrants prospective validation in larger, multi-center cohorts.

## 1 Background

The World Health Organization (WHO) declared COVID-19 a global public health emergency on January 30, 2020 (1). Following China’s lifting of epidemic control measures on December 7, 2022, a surge in severe COVID-19 cases was observed, with mortality rates escalating despite intensive care (2). This resurgence highlighted critical gaps in predicting treatment outcomes, particularly for immunomodulatory therapies like glucocorticoids.

During infection, COVID-19 triggers complex immune cascades ranging from hyperinflammation to immunosuppression (3). While biomarkers such as neutrophil-to-lymphocyte ratio (NLR) and C-reactive protein (CRP) are widely used for severity assessment (4), they show poor specificity in guiding targeted therapies – a multicentre cohort study revealed CRP’s limited prognostic value for glucocorticoid non-responders (Area under the curve (AUC) = 0.61, sensitivity 66%, specificity 59%) (5). Recent evidence suggests that both ferritin and IL-10 serve as significant predictors for certain health conditions. Specifically, a serum ferritin >3,000 μg/L not only indicates macrophage activation severity (6) but also stratifies Acute Respiratory Distress Syndrome (ARDS) risk (Odds ratio (OR) = 5.2, *p* < 0.001) (7). Additionally, IL-10 elevation >20 pg./mL correlates with Treg-mediated immunosuppression and glucocorticoid resistance (8).

Glucocorticoids have been used for over half a century to alleviate cytokine storms in severe infections and to treat ARDS (9). Although the RECOVERY trial established dexamethasone’s mortality benefit in severe COVID-19, subsequent meta-analyses reveal significant response heterogeneity across subgroups (10). In China, the Novel Coronavirus Diagnosis and Treatment Protocol (10th edition) recommends short-term glucocorticoid use for patients with severe novel coronavirus infection. Nevertheless, about 30–40% of patients show suboptimal response, highlighting the urgent need for predictive biomarkers to guide personalized therapy (11). This underscores the inadequacy of current “one-size-fits-all” approaches.

To address this precision medicine gap, we used common laboratory indicators from appropriate patients at admission to develop an effective and visual prognostic model to identify, at admission, patients at high risk of poor clinical outcomes despite receiving glucocorticoid therapy. The model’s primary outcomes were hospital discharge, death, or clinical improvement, assessed through rigorous discrimination and calibration analyses.

## 2 Materials and methods

### 2.1 Study design and participants

This retrospective study included patients with severe COVID-19 (defined by the WHO criteria: oxygen saturation ≤94% on room air or requiring high-flow oxygen/non-invasive ventilation) from December 1, 2022, to August 20, 2023. The diagnosis was confirmed via nucleic acid amplification assays [e.g., reverse transcription-polymerase chain reaction (RT-PCR)] in respiratory specimens, following both the Diagnosis and Treatment Scheme of Pneumonia Caused by Novel Coronavirus of China (10th edition) and WHO guidelines (WHO, 2020). The exclusion criteria included: (1) no glucocorticoid therapy; (2) immunocompromised status (e.g., HIV, active chemotherapy); (3) incomplete laboratory/imaging data; (4) death from non-respiratory causes. The study was approved by the Ethics Review Committee of the First Hospital of Jilin University and adhered to the principles outlined in the Declaration of Helsinki. Given the retrospective design of the study, the requirement for written informed consent was waived.

### 2.2 Data collection

All patients received glucocorticoids within 7 days of symptom onset. The non-response group (*n* = 51) received methylprednisolone at an initial dose of 80 mg/day, tapered based on clinical response (maximum 10 days). This regimen aligns with the WHO-recommended protocol for severe COVID-19 (WHO, 2020) (12) and previous trials using methylprednisolone in ARDS (13). Within 72 h of glucocorticoid administration, the dosage of the hormone was initially adjusted based on the oxygenation index and patient symptoms. The specific dosage adjustment was determined by the clinician. The maximum hormone dosage in this study centre was 240 mg/d. Clinical information, including sex, age, comorbidities (hypertension, diabetes, heart disease, cerebral infarction, and immunosuppressive use), and laboratory indicators, were retrospectively collected. Treatment failure (non-response group) was defined as meeting ≥1 criteria during hospitalization: (1) The primary outcome was defined as death from respiratory failure or escalation to invasive mechanical ventilation/ECMO. (2) Lung CT [extensive bilateral exudation, according to Asemi-quantitative scoring system developed by the British Thoracic Imaging Society (14)]. (3) Persistent organ dysfunction. (4) Inflammation indicators (e.g., CRP) exceeding the normal range with continued elevation. For the purpose of this study, criteria (1) was considered the primary determinants of treatment failure.

### 2.3 Data analysis

The collected data were classified into continuous and categorical variables. All laboratory indicators were considered continuous variables. Normally distributed continuous variables were expressed as mean ± standard deviation (SD) and compared using the unpaired T-test. Continuous variables with a skewed distribution were expressed as median (P25, P75) and analysed using the Wilcoxon signed-rank test. Categorical variables were expressed as frequencies (percentage, %) and compared using Pearson’s chi-squared test. The final enrolled patients were randomly divided into training and test cohorts in a 7:3 ratio. R version 4.4.1 was used for statistical analysis and graphic production. To address missing data, variables with <20% missing values were imputed using multiple imputation by Chained Equations (*MICE*). To verify the impact of data imputation on the final results, we conducted a sensitivity analysis on the data. That is, using data without missing values, we verified whether ferritin, IL-10, and diabetes were still independent risk factors, and also verified the AUC value of the model. For biomarker cut-off determination, receiver operating characteristic (ROC) curves were generated. Optimal thresholds were selected by maximizing Youden’s index (sensitivity + specificity − 1). The final cohort was randomly split into training and test sets (7:3 ratio) to validate the model. All analyses were conducted in R software (version 4.4.1; R Foundation) with packages including pROC (v1.18.4) for receiver operating characteristic (ROC) analysis and mice (v3.16.0) for missing data imputation. The 10-fold cross-validation was stratified by the outcome to preserve the event-to-non-event ratio in each fold. All preprocessing steps were applied within training folds to avoid data leakage (caret in R 4.41). Diagnostic accuracy parameters and their exact confidence intervals were computed using the epiR package (15) in R. A two-sided *p* < 0.05 was considered statistically significant.

### 2.4 Prediction model building

The prediction model was developed in two steps (16). First, univariate logistic regression was used to screen variables with *p* < 0.05 while plotting the ROC curve and calculating the AUC for each variable. Multicollinearity was evaluated using Spearman’s correlation heatmap and variance inflation factor (VIF). The heatmap highlighted strong pairwise correlations. VIF analysis revealed severe multicollinearity in each variable. This combined approach balanced visual correlation analysis with robust VIF quantification. Least absolute shrinkage and selection operator (LASSO) regression was performed to analyse variations in mean squared error and variable coefficients based on *λ* values, effectively mitigating collinearity and overfitting. In LASSO regression, *λ*.min was defined as the *λ* value corresponding to the lowest mean squared error, while λ.1se was defined as a 1-standard error to the right of λ.min. When λ.1se is used, variables with non-zero coefficients in LASSO regression were selected for multifactorial logistic regression. To evaluate the model, discrimination and calibration were assessed using two aspects. Discrimination and calibration were evaluated using not only the AUC of the ROC curve and a calibration curve, but also various accuracy measures, including sensitivity, specificity, false positive and negative rates, positive and negative predictive values, Jorden index, accuracy, positive and negative likelihood ratios, and diagnostic odds ratios. These measurements and their precise confidence intervals are critical to determining diagnostic performance. Finally, a clinically applicable nomogram was constructed and used to calculate the total score for each patient.

## 3 Results

### 3.1 Baseline characteristics

**Figure 1:**
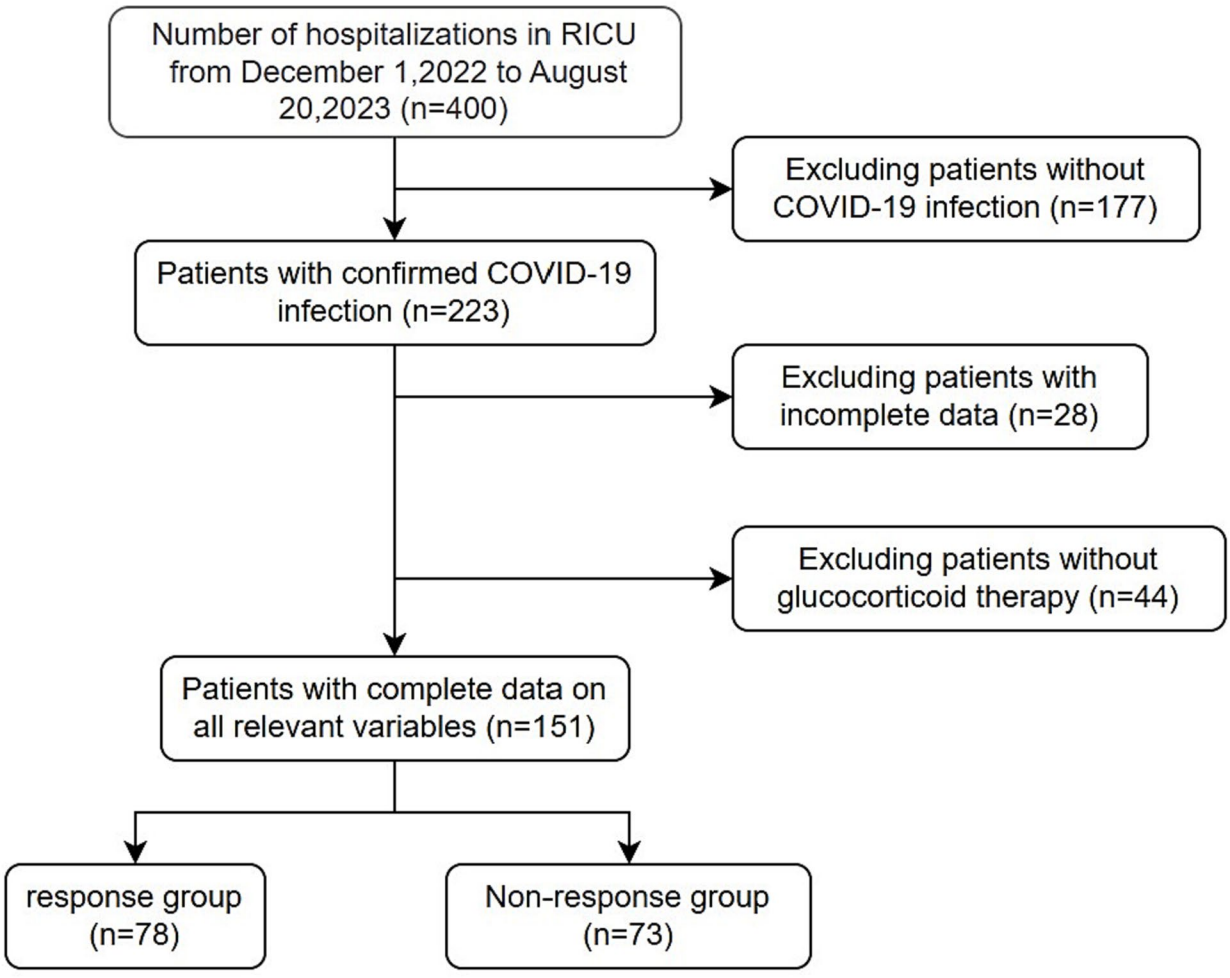
Flow chart of patients’ final diagnosis and enrollment.

Medical records and laboratory data were obtained from 400 patients with COVID-19, all over 18 years old and treated with glucocorticoids. Ultimately, 151 patients were included in the study based on the exclusion criteria (Figure 1). The median age (IQR) of the 151 enrolled patients was 72 (65–79) years. The baseline patient characteristics are presented in Table 1. The non-response cohort was older, more likely to have diabetes, and had higher levels of D-D polymers, Pro-BNP, direct bilirubin, and inflammatory markers, including ferritin, C-reactive, procalcitonin, IL-6, IL-10, and IL-17A than the response cohort. They also had lower platelet, lymphocyte, and albumin levels. Additionally, patients who died had more pronounced symptoms of dyspnoea at the onset of infection.

**Table 1:** Baseline characteristics of patients.

Variables	Reference range	Response group (*n* = 78)	Non-response group (*n* = 73)	*p*-value
Sex, *n* (%)				0.742
Female		33 (42)	28 (38)	
Male		45 (58)	45 (62)	
Hypertension, *n* (%)		38 (49)	39 (53)	0.678
Diabetes, *n* (%)		15 (19)	28 (38)	0.015
Cardiovascular disease, *n* (%)		18 (23)	20 (27)	0.672
Cerebral infarction, *n* (%)		10 (13)	6 (8)	0.513
Fever, *n* (%)		66 (85)	61 (84)	1
Cough, *n* (%)		66 (85)	63 (86)	0.95
Expectoration, *n* (%)		65 (83)	62 (85)	0.964
Dyspnea, *n* (%)		47 (60)	59 (81)	0.01
Chest pain, *n* (%)		1 (1)	0 (0)	1
Hemoptysis, *n* (%)		3 (4)	3 (4)	1
Time from symptom onset (days)		7 (5, 10)	7 (5, 8)	0.074
Age (years)		71 (62.25, 78)	74 (68, 79)	0.043
Temperature (°C)		38.2 (37.52, 39)	38 (36.9, 38.5)	0.133
Respiratory rate		20 (20, 22)	20 (20, 23)	0.147
Pulse		84.5 (78, 92)	88 (80, 106)	0.089
SBP (mmHg)		134.73 ± 19.91	135.95 ± 21.27	0.718
DBP (mmHg)		78 (71, 86)	78 (70, 83)	0.599
WBC (×10^9^/L)	3.5–9.5	8.43 (5.34, 10.91)	9.19 (5.61, 12.75)	0.291
NE (×10^9^/L)	1.8–6.3	7.15 (4.6, 9.73)	8.45 (4.42, 11.66)	0.164
LY (×10^9^/L)	1.1–3.2	0.64 (0.39, 0.88)	0.44 (0.31, 0.58)	< 0.001
MO (×10^9^/L)	0.1–0.6	0.41 (0.27, 0.66)	0.38 (0.23, 0.57)	0.544
HB (g/L)	120–150	128 (121.25, 141.5)	131 (114, 146)	0.889
PLT (×10^9^/L)	125–350	205.5 (154.5, 272.75)	175 (123, 248)	0.051
AST (U/L)	13–35	31.05 (22.02, 45.03)	32 (23.8, 52.1)	0.416
ALT (U/L)	7–40	30.4 (18.45, 49.33)	27.2 (18, 40.8)	0.214
γ-GT (U/L)	7–45	55.45 (33.78, 128.48)	55.9 (35.9, 109.5)	0.97
ALB (g/L)	40–55	32 (29.72, 34.65)	30.2 (27.4, 31.9)	0.001
GLB (g/L)	20–40	28.35 ± 4.32	29.12 ± 5.94	0.368
A/G	1.2–2.4	1.16 (1.03, 1.25)	1.06 (0.91, 1.25)	0.024
TBIL (umol/L)	0–21	11.95 (9.33, 15.7)	13.7 (10, 17.1)	0.092
DBIL (umol/L)	0–6.8	2.75 (1.9, 4.3)	3.7 (2.4, 5.6)	0.012
IBLI (umol/L)	5–20	9.3 (7, 11.07)	9.8 (7.3, 12.1)	0.235
TBA (umol/L)	0–10	3.45 (2.1, 4.88)	3.8 (2.3, 6.9)	0.242
BUN (mmol/L)	2.6–7.5	5.92 (4.81, 7.91)	6.73 (5.34, 9.46)	0.079
CR (umol/L)	41–73	59.2 (48.5, 76.53)	62.5 (50.9, 89.2)	0.248
CRP (mg/L)	0–1	60.76 (28.03, 117.26)	92.7 (53.25, 173.14)	0.011
PCT (ng/L)	0–0.5	0.06 (0.05, 0.28)	0.16 (0.05, 1.55)	0.001
Pro-BNP (pg/mL)	0–125	483.5 (162.5, 859.58)	765 (253, 2,610)	0.009
FET (ug/L)	23.9–336.2	807.05 (521.02, 1050.28)	1327.4 (939.1, 1897.4)	< 0.001
D-D (mg/L FEU)	0–0.5	0.82 (0.4, 1.22)	1.73 (0.88, 8.18)	< 0.001
FDP (ug/mL)	0–5	2.9 (2.5, 4.64)	5.8 (3.39, 22.28)	< 0.001
APTT (s)	21–33	28.5 (26.58, 30.7)	28.6 (26, 30.3)	0.898
PT (s)	9–13	11.8 (11.3, 12.28)	12.4 (11.6, 13.5)	< 0.001
INR	0.8–1.2	1 (0.94, 1.07)	1.06 (0.99, 1.17)	< 0.001
IL-2 (pg/mL)	0–5.71	2.54 (1.99, 2.98)	2.38 (1.93, 3.85)	0.463
IL-4 (pg/mL)	0–2.8	2.46 (1.93, 3.26)	2.63 (2, 4.3)	0.148
IL-6 (pg/mL)	0–5.3	31.41 (9.91, 70.76)	91.04 (31.46, 402.08)	< 0.001
IL-10 (pg/mL)	0–4.9	4.08 (2.68, 6.92)	7.53 (4.8, 13.84)	< 0.001
TNF-α (pg/mL)	0–2.31	2.21 (1.63, 2.76)	2.34 (1.81, 2.72)	0.445
Interferon-γ (pg/mL)	0–7.42	2.94 (2.16, 5.42)	2.85 (2.19, 3.93)	0.496
IL-17A (pg/mL)	0–20.6	4.6 (2.41, 10.41)	8.9 (4.44, 17.16)	0.005

### 3.2 Risk factors for hormonal therapy failure

**Figure 2:**
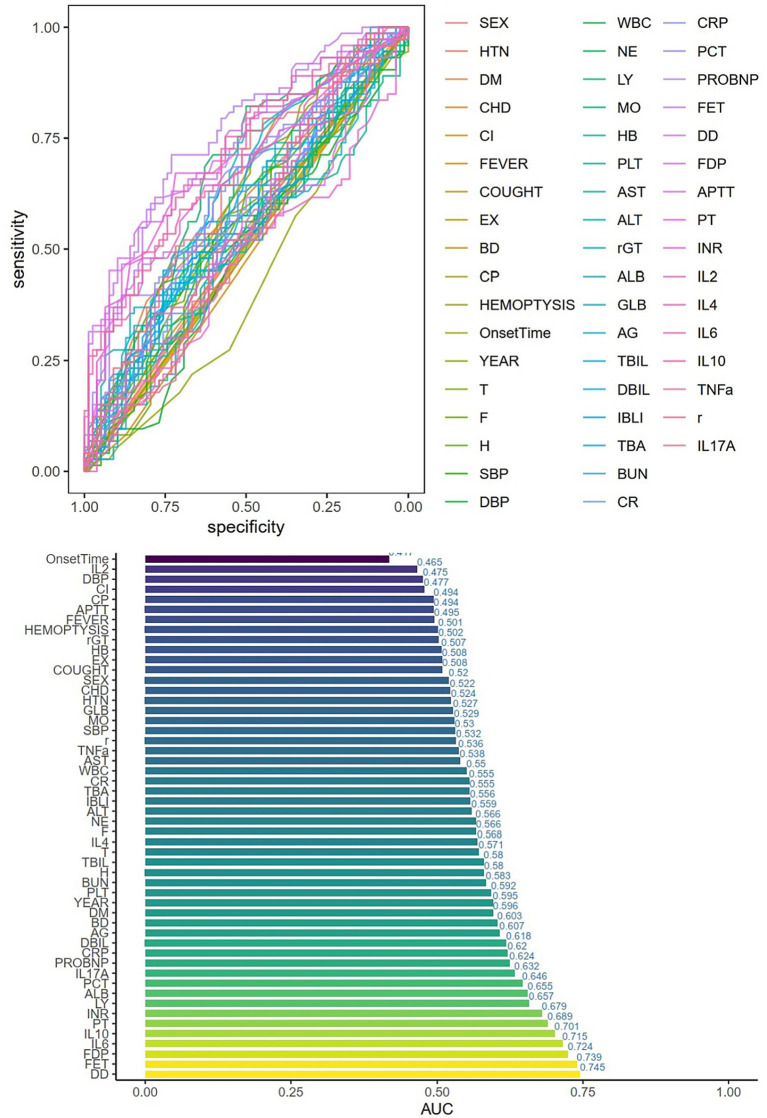
Single-factor logistic regression and AUC values for each variable. HTN = high blood pressure, DM = Diabetes, CHD=Cardiovascular disease, CI=Cerebral infarction, EX = Expectoration, BD=Dyspnea, CP=Chest Pain, T = Temperature, F = Respiratory rate, H=Pulse, SBP = systolic blood pressure, DBP = diastolic blood pressure, WBC = white blood cell, NE = Neutrophils, LY = lymphocytes, MO = monocytes, HB = hemoglobin, PLT = platelets, AG = albumin/globulin, TBA = total bile acid, BUN = urea nitrogen, CR = creatinine, CRP=C-reactive protein, PCT = procalcitonin, FER = ferritin, r = IFN-*γ*.

**Figure 3:**
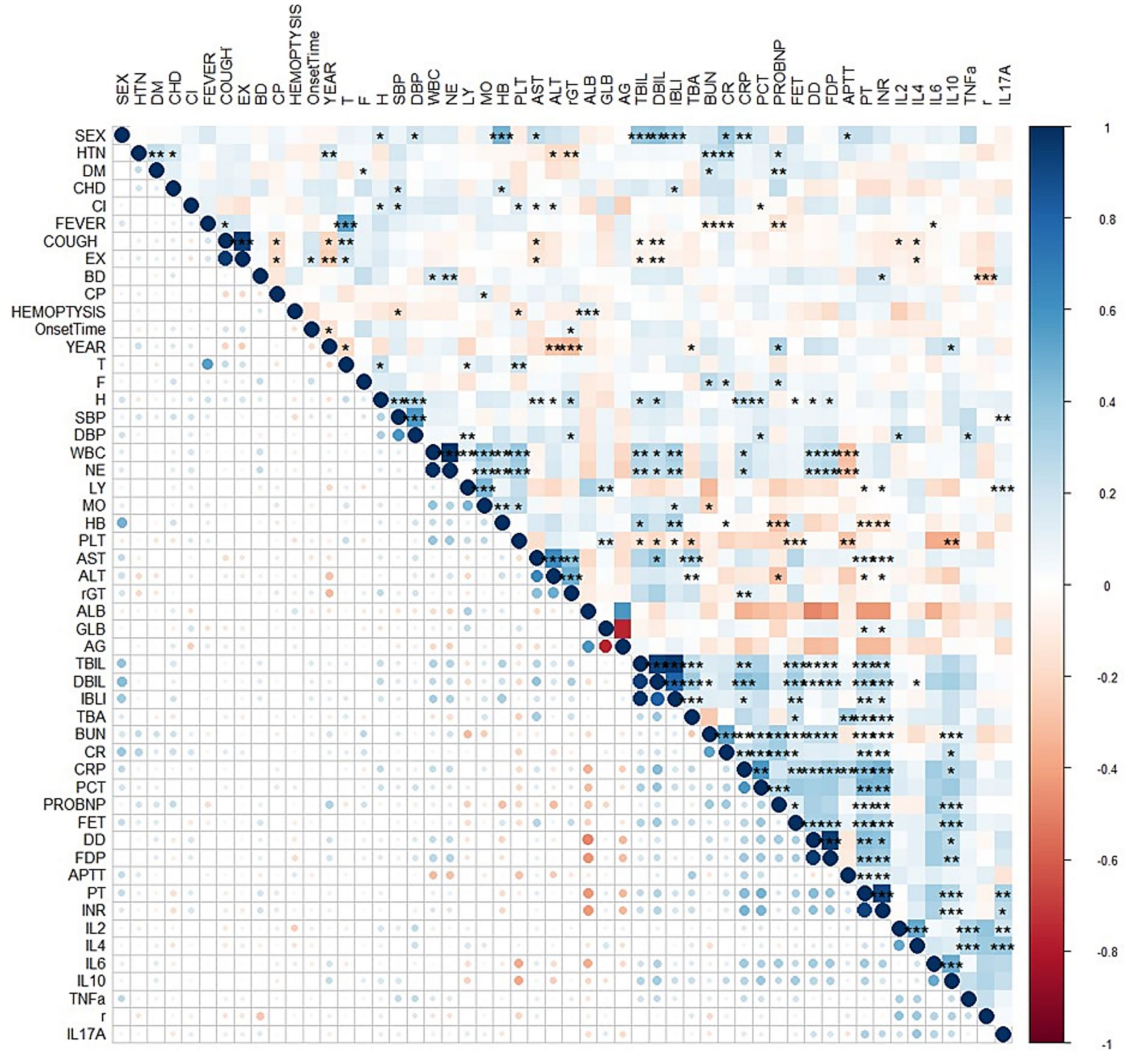
Correlation analysis between variables represented by a heatmap. HTN = high blood pressure, DM = Diabetes, CHD=Cardiovascular disease, CI=Cerebral infarction, EX = Expectoration, BD=Dyspnea, CP=Chest Pain, T = Temperature, F = Respiratory rate, H=Pulse, SBP = systolic blood pressure, DBP = diastolic blood pressure, WBC = white blood cell, NE = Neutrophils, LY = lymphocytes, MO = monocytes, HB = hemoglobin, PLT = platelets, AG = albumin/globulin, TBA = total bile acid, BUN = urea nitrogen, CR = creatinine, CRP=C-reactive protein, PCT = procalcitonin, FER = ferritin, r = IFN-γ.

**Figure 4:**
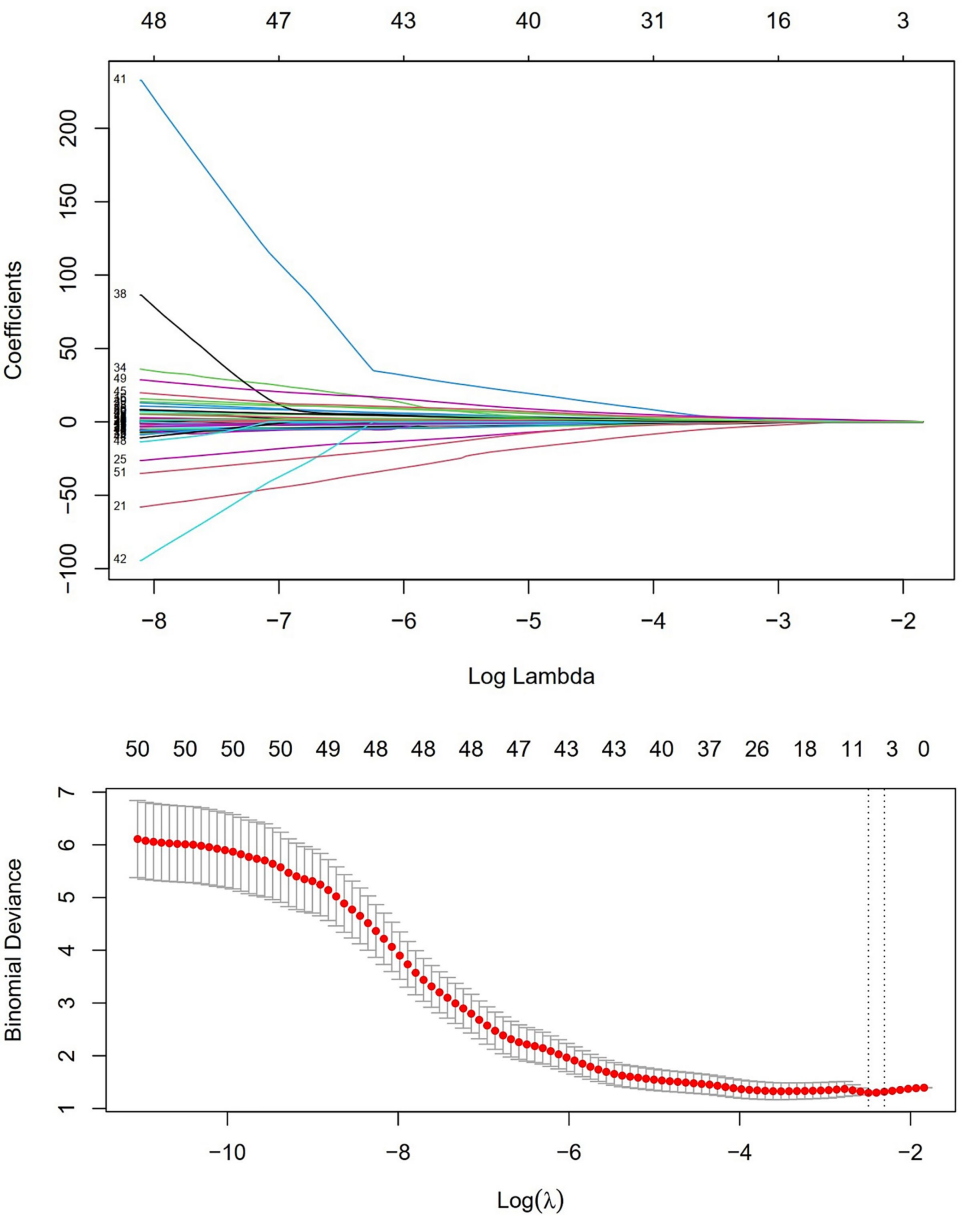
LASSO regression and independent risk factor selection.

**Figure 5:**
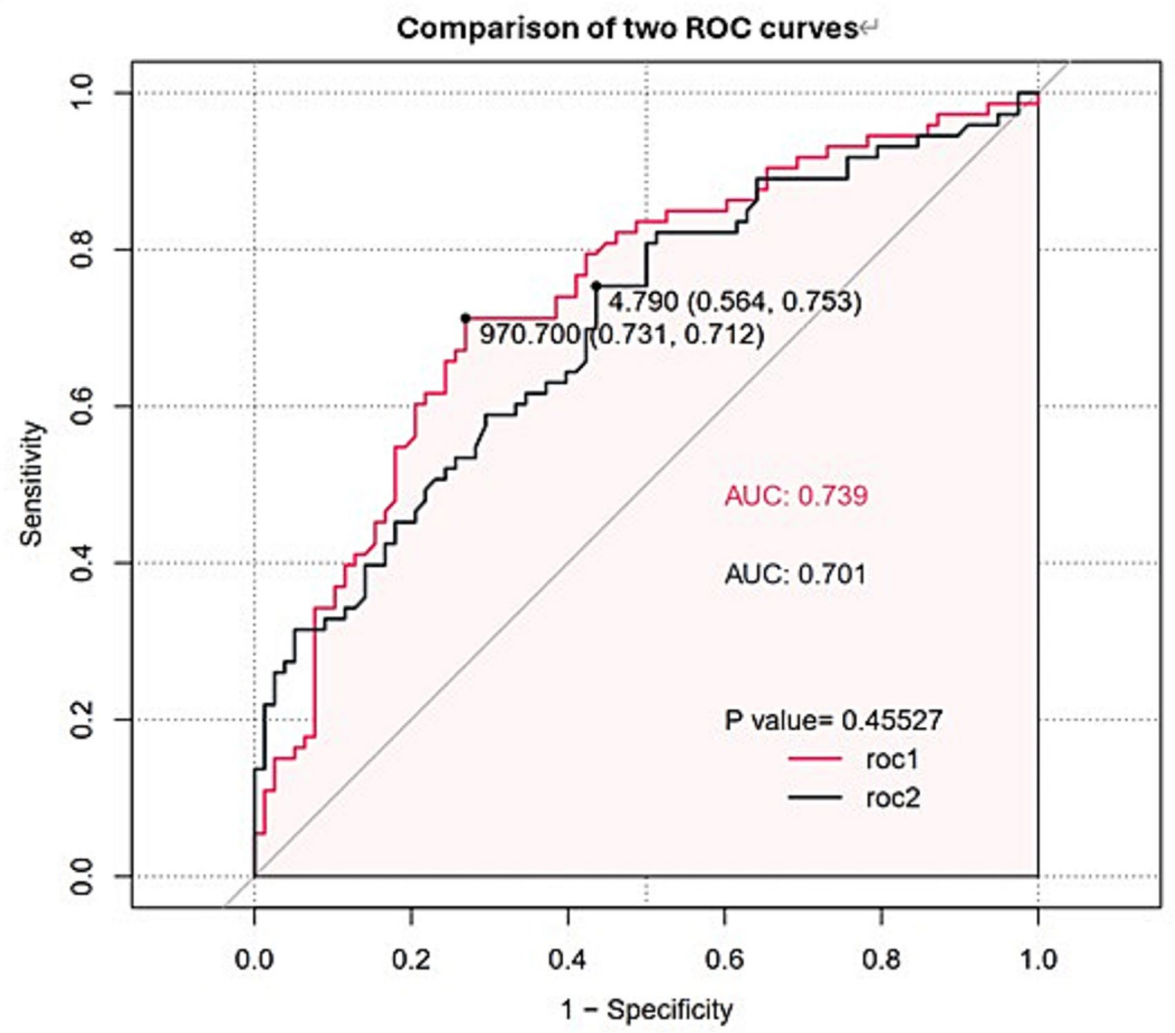
ROC curves and AUC values of FET and IL-10. The red line (ROC1) represents FET, while the black line (ROC2) represents IL-10.

Univariate logistic regression was conducted to assess the AUC for each variable (Figure 2). Correlations between variables were then analysed (Figure 3; Supplementary Table 1), and multicollinearity was addressed using LASSO regression. After univariate analysis and LASSO regression (Figure 4), diabetes, dyspnoea, FET, IL-10, FDP, INR, and Pro-BNP were included in the multifactorial analysis. The prediction model incorporated diabetes, FET, and IL-10 as the variables. The optimum cut-off values for ferritin and IL-10 were determined using the Youden index, with ferritin at 970.7ug/L and IL-10 at 4.79 pg./mL (Figure 5). A sensitivity analysis confined to complete cases (*n* = 118) yielded consistent results, with ferritin and IL-10 remaining significant predictors (all *p* < 0.05) in Supplementary Table 4. The AUC results of the model established based on the complete data (AUC = 0.78) are roughly the same as those of the model trained on the original training set (AUC = 0.779).

### 3.3 Subgroup analysis of ferritin in patients with severe COVID-19

To further characterize the clinical significance of elevated ferritin, we stratified the cohort based on the optimal cut-off value of 970.7 ng/mL. The hyperferritinemic group showed significantly elevated inflammatory markers (CRP, PCT, IL-6; all *p* < 0.01), more pronounced coagulopathy (higher D-dimer and FDP, *p* < 0.001), and greater evidence of hepatic and renal dysfunction (elevated AST, TBIL, and Cr; *p* < 0.05) compared to those with lower ferritin levels. This identifies a distinct high-risk subgroup characterized by enhanced systemic inflammation and multi-organ involvement.

### 3.4 Construction of the prediction model and nomogram

**Figure 6:**
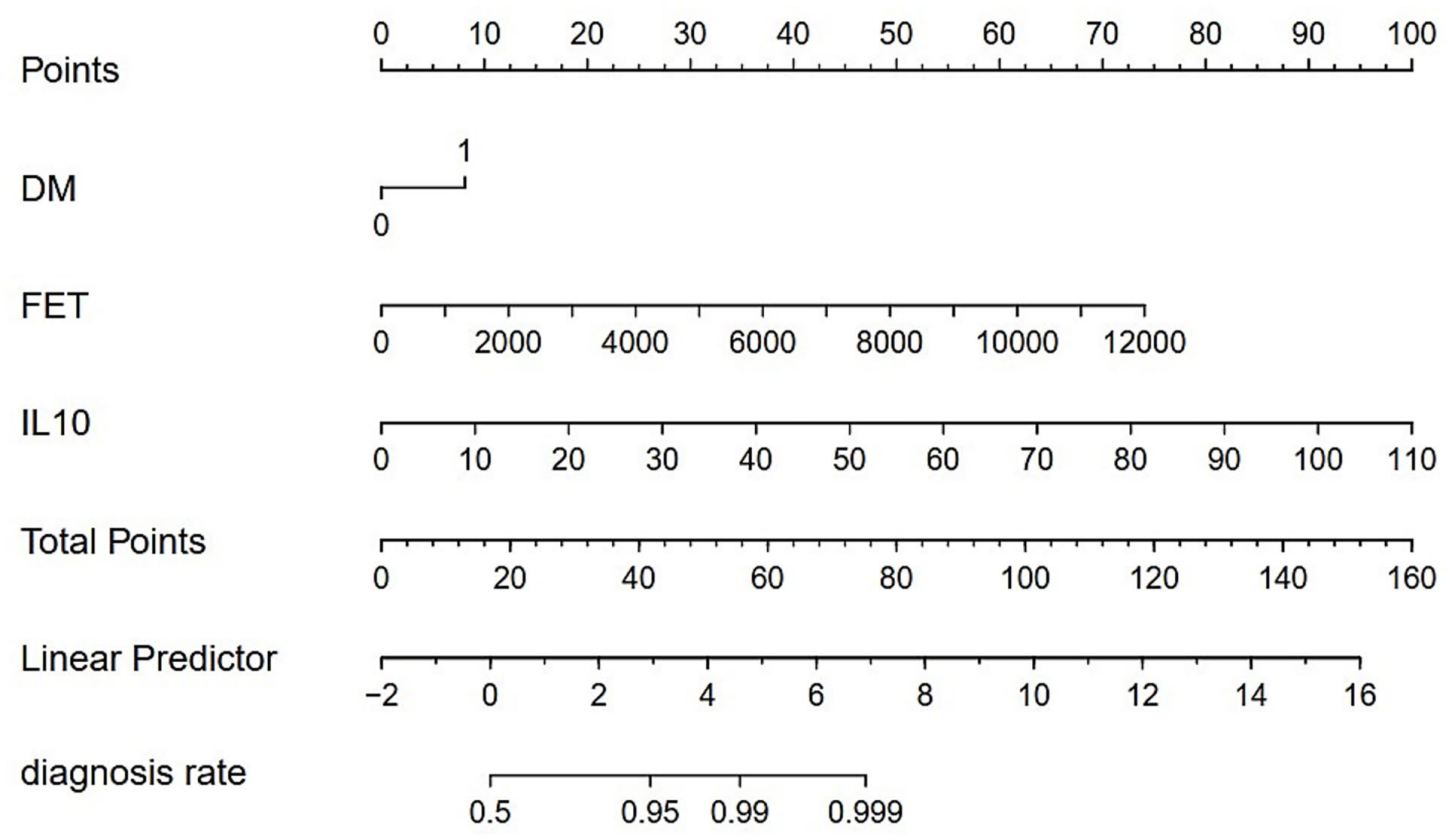
Nomogram for clinical prediction. DM = Diabetes, FET = Serum Ferritin, IL-10 = Interleukin-10. 0 = Non-diabetic; 1 = Diabetic.

**Figure 7:**
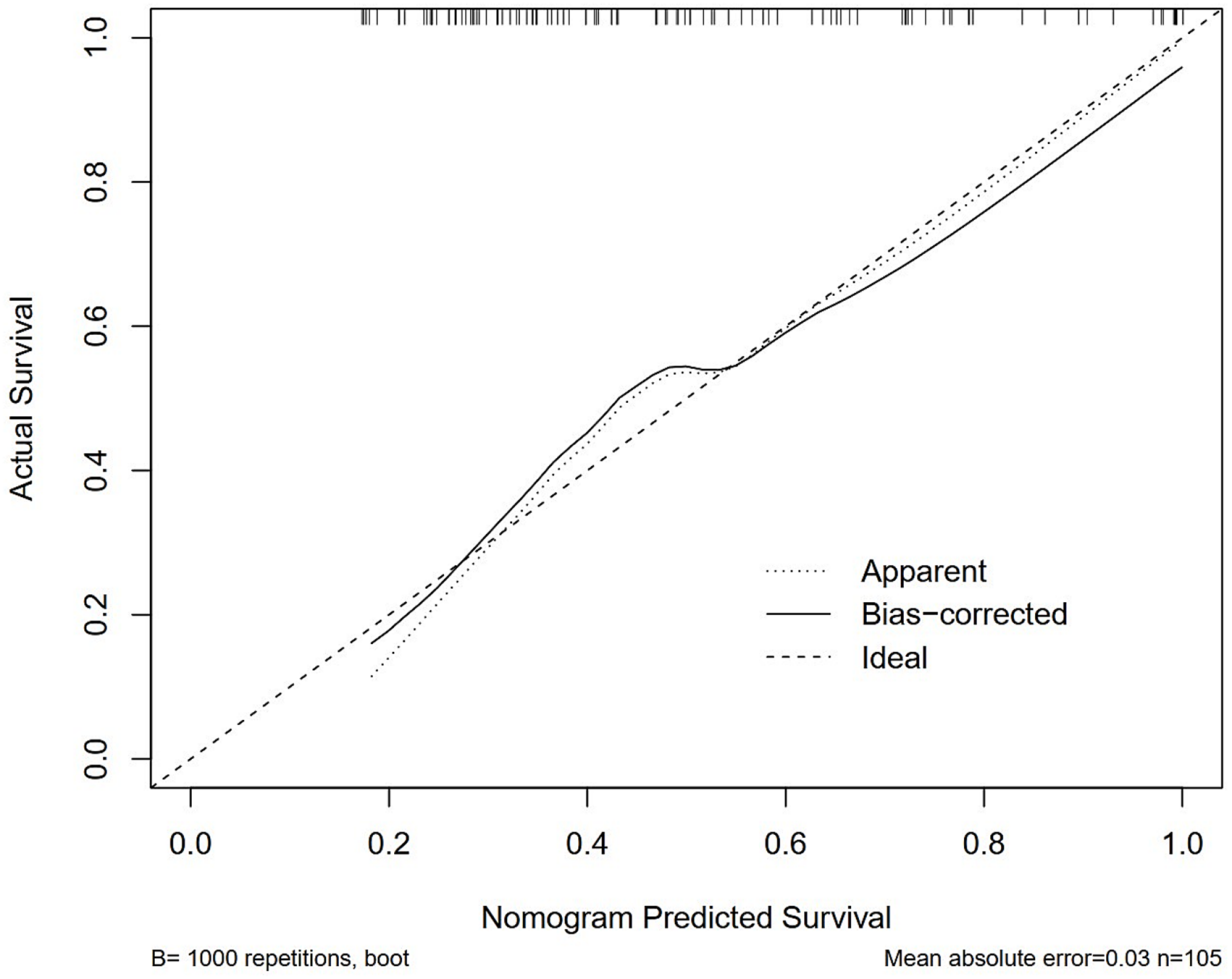
Calibration curves of the nomogram in the training cohort. Hosmer–Lemeshow test: *p* > 0.05, indicating good model fit.

Logistic regression analysis was performed to calculate the OR of candidate variables and construct the predictive model (Table 2). A nomogram was developed as a visual predictive tool for clinical application (Figure 6). Key variables (ferritin, IL-10, diabetes) were assigned scores based on their regression coefficients, with total scores mapped to predicted probabilities. For example, a 70-year-old patient with diabetes (score = 10), ferritin = 5,000 μg/L (score = 30), and IL-10 = 20 pg./mL (score = 20), the total score (60 points) corresponds to a 84.7% risk of treatment failure (95% confidence interval (CI): 65–92%). The model demonstrated robust discriminative ability, with a training set C-index of 0.779 (95% CI: 0.69–0.86) and test set C-index of 0.780 (95% CI: 0.64–0.92). Comprehensive diagnostic performance metrics were calculated using the R package epiR (v2.0.50) at the optimal probability threshold (0.5). The model achieved a sensitivity of 71.4% (95% CI: 59.4–81.6%), specificity of 70.0% (95% CI: 60.0–78.8%), and accuracy of 70.6% (95% CI: 63.1–77.3%). Further metrics included a positive predictive value (PPV) of 62.5% (95% CI: 51.0–73.1%), negative predictive value (NPV) of 77.8% (95% CI: 67.8–85.9%), and Youden’s index of 0.414. The positive likelihood ratio (LR+) was 2.38 (95% CI: 1.70–3.33), the negative likelihood ratio (LR−) was 0.41 (95% CI: 0.28–0.60), and the diagnostic odds ratio (DOR) reached 5.83 (95% CI: 2.98–11.42) (Supplementary Table 2). Given the moderate sample size (*n* = 151), 10-fold cross-validation was prioritized, yielding a mean AUC of 0.755 (SD = 0.189), which is slightly lower than the initial split-sample test AUC (0.780). Despite the variability reflected in the SD, the cross-validated AUC demonstrates reasonable generalizability in predicting corticosteroid efficacy. Calibration analysis confirmed excellent agreement between predicted and observed outcomes: Hosmer-Lemeshow test χ^2^ = 4.2 (*p* = 0.84) (Figure 7), with a Brier score of 0.182 and calibration slope of 0.95 (95% CI: 0.82–1.08). Cross-validation further demonstrated stable performance across subsets (mean absolute error = 0.03, mean squared error = 0.00127).

**Table 2:** Predictors for the nomogram.

Intercept and variable	β	Odds ratios (95% CI)	*P*-value
Intercept	−2.004		0.0002
DM	0.9608	2.61 (0.961, 7.108)	0.0597
FET	0.0007	2.10 (1.077, 4.096)	0.0294
IL-10	0.1077	1.89 (1.189, 3.206)	0.0174
C-index			
Training cohort	0.779 (0.69–0.86)		
Test cohort	0.780 (0.64–0.92)		

## 4 Discussion

This single-centre retrospective study found significant differences in the immunomodulatory effects of glucocorticoids in patients with severe COVID-19 pneumonia. This heterogeneity in treatment response exists within the established context that systemic corticosteroids overall reduce mortality in severe COVID-19, as unequivocally demonstrated by large-scale trials like RECOVERY and subsequent meta-analyses. However, our study identifies ferritin and IL-10 as pivotal biomarkers for predicting glucocorticoid responsiveness in severe COVID-19, addressing a critical gap highlighted by the RECOVERY trial’s subgroup heterogeneity (10). When ferritin and IL-10 levels exceeded 970.7 ng/mL and 4.79 pg./mL, respectively, glucocorticoid therapy was ineffective in regulating immune responses. Therefore, glucocorticoids should not be used in a one-size-fits-all manner in patients with severe COVID-19. Several studies have suggested that the ineffectiveness of glucocorticoid therapy in COVID-19 may be related to cytokine storm, which triggers and sustains a systemic hyperinflammatory response. This inflammatory response ultimately leads to ARDS, multi-organ failure, and even death (17). Although the precise mechanism of lung injury remains unclear, cytokine storm is widely recognized as an essential contributor (18). Recent randomized controlled trials and meta-analyses have shown that methylprednisolone reduces systemic inflammatory responses and shortens the duration of mechanical ventilation in patients with ARDS (19). However, clinical interventions for COVID-19 patients who have entered the cytokine storm phase demonstrate varying success rates depending on the timing and patient selection (20). Based on the observed variability in glucocorticoid treatment outcomes, we hypothesized that the use, timing, and dosage of glucocorticoids should be evaluated based on serum biomarkers in patients with severe COVID-19.

The clinical manifestations of severe COVID-19 resemble ferritin syndromes, including systemic inflammatory response syndrome (SIRS) and ARDS (21). Patients with COVID-19 exhibit markedly elevated ferritin levels, lymphopenia, reduced NK cells and activity, abnormal liver function tests, and coagulation disorders, making COVID-19 the newest member of the ferritin syndrome group (22). Extreme hyperferritinemia (>970.7 ng/mL in our cohort) is not merely a biomarker but a central feature of uncontrolled macrophage activation and cytokine storm (6). This state of innate immune activation may paradoxically lead to glucocorticoid treatment failure. From a basic science standpoint, this resistance can be explained by the interplay between hyperinflammatory signaling and the glucocorticoid receptor (GR). Sustained activation of pattern recognition receptors (e.g., TLRs) on macrophages leads to relentless activation of pro-inflammatory transcription factors like NF-κB and AP-1 (23). These transcription factors can physically interfere with GR function through protein–protein interactions, a mechanism known as “cross-talk”, thereby impairing the GR’s ability to transrepress inflammatory genes (24). Furthermore, inflammatory cytokines can activate kinases (e.g., p38 MAPK, JNK) that phosphorylate the GR, altering its conformation, reducing its ligand-binding affinity, and promoting its nuclear export (25). This effectively blunts the anti-inflammatory action of glucocorticoids at a molecular level. Our finding aligns with the findings of Zhou et al. (7). Besides, studies indicate that elevated ferritin levels may increase the risk of ARDS or poor prognosis in COVID-19 (26). Therefore, reducing ferritin levels following glucocorticoid therapy in patients with severe COVID-19 will help mitigate inflammatory response and improve prognosis. Notably, high-dose methylprednisolone (>125 mg/day) improved outcomes in hyperferritinemic patients (>500 ng/mL) (27), yet our cohort received lower doses (80 mg/day), which may explain the high rate of therapeutic failure observed in our patients whose ferritin levels exceeded our derived cut-off value of 970.7 ng/mL. This underscores the need for biomarker-guided dosing escalation. Low to moderate glucocorticoid doses may fail to adequately control hyperinflammation, while such doses could suppress immune response through multiple pathways, potentially impairing viral clearance. Therefore, in patients with COVID-19, careful consideration of timing and dosage is essential when administering glucocorticoids. Furthermore, patients with ferritin levels above 970.7 ng/mL constituted a distinct subgroup with markedly elevated inflammation, coagulopathy, and evidence of organ dysfunction, which may explain the failure of moderate-dose glucocorticoids to control such a severe hyperinflammatory state. The relationship between ferritin levels and glucocorticoid efficacy remains unknown; further prospective studies are needed to validate these findings.

This study also found that IL-10 elevation at the time of admission could be a predictor of glucocorticoid therapy efficacy. Elevated IL-10 (>4.79 pg./mL) marked glucocorticoid resistance, consistent with Chen et al.’s findings that IL-10 > 15 pg./mL predicted mortality (adjusted HR = 2.01) in severe COVID-19 (28). We speculate that several mechanisms could explain these findings: first, IL-10 is an anti-inflammatory cytokine secreted at sites of persistent inflammation to maintain a balance between effective pathogen clearance and immune regulation (29). However, many pathogens induce IL-10 upregulation during infection and exploit its immunosuppressive activity to evade the host immune system (30), potentially promoting a microenvironment conducive to their persistence and survival. Glucocorticoids have been shown to reduce systemic inflammation in severe COVID-19, particularly during the intense inflammatory phase after the initial viral replication stage. However, at the same time, it also impairs viral clearance (31), and this combined effect may aggravate patient outcomes. Second, as a key immunoregulatory cytokine, IL-10 is upregulated during intense inflammation, serving as a critical feedback mechanism to counteract potential tissue damage from excessive proinflammatory cytokine release. It is produced by a diverse range of immune cells—such as natural killer (NK) cells, dendritic cells, monocytes, and macrophages—to exert its potent anti-inflammatory and immunosuppressive effects (32). Increased IL-10 secretion reflects an enhanced immune response to viral infection. However, glucocorticoid use further suppresses the body’s immune response, weakening viral clearance and increasing the risk of mortality. Lastly, IL-10 elevation may signal Treg exhaustion and secondary HLH-like syndromes (33), creating an systemic immunosuppression that glucocorticoids cannot reverse. Furthermore, our finding that elevated baseline IL-10 predicts glucocorticoid resistance presents an apparent paradox, given that IL-10 is itself a glucocorticoid-response gene and its expression is known to be upregulated by glucocorticoid therapy (34). This suggests that the pre-treatment elevation of IL-10 in non-responders may originate from a different, potentially dysregulated pathway. This could be influenced by host genetic factors. Single nucleotide polymorphisms (SNPs) in the promoter region of the *IL10* gene can significantly alter its basal transcriptional activity (35). Likewise, SNPs in the glucocorticoid receptor gene (*NR3C1*) could impair receptor function and downstream anti-inflammatory signaling, while potentially leaving some transcriptional activities like IL-10 induction intact (36). Thus, non-responders might possess a genetic predisposition that favors high baseline IL-10 production—marking a state of immune imbalance that is paradoxically less responsive to exogenous glucocorticoids—or a GR variant that fails to adequately suppress inflammation despite activating certain genes like IL10. This contrasts with IL-10’s early protective role in balancing inflammation (37), highlighting its context-dependent duality. These findings suggest that IL-10 levels at admission in patients with COVID-19 may be a valuable biomarker for early disease prediction, identifying higher-risk patients prone to severe complications and determining the most appropriate treatment regimen and duration (38). Meanwhile, our findings extend Del Valle et al.’s work (6), suggesting IL-10 thresholds could identify patients requiring alternative immunomodulators (e.g., JAK inhibitors) instead of glucocorticoids.

In addition, the effectiveness of glucocorticoids in patients with diabetes and severe COVID-19 may be limited. Some studies have reached conclusions similar to ours, showing that people with diabetes have a higher mortality rate from COVID-19. Furthermore, dexamethasone, a potent glucocorticoid used for moderate to severe COVID-19, may exacerbate hyperglycaemia in patients with diabetes, potentially leading to worse outcomes (39). Diabetes mellitus is associated with a proinflammatory state and attenuated innate immune responses. Metabolic disorders may impair macrophage and lymphocyte function, leading to immune dysfunction and increasing susceptibility to disease complications (40). Compared to patients with COVID-19 who do not have diabetes, those with diabetes had heightened inflammatory responses (characterized by elevated neutrophils, serum ferritin, IL-6, CRP, and erythrocyte sedimentation rate) and suppressed immunity (marked by significantly reduced lymphocyte levels) (41). These findings suggest that patients with COVID-19 and diabetes may be more susceptible to excessive inflammation and immune dysregulation. Given the retrospective nature of our study and the timing of glucocorticoid administration, further prospective, randomized controlled studies are needed to better understand these effects.

In summary, this dual-axis approach aligns with emerging frameworks for COVID-19 endo-typing (42), which emphasize the biphasic immunopathology of early hyperinflammation and late immune exhaustion. Corticosteroid effects vary significantly depending on the host’s immune response. We have clarified in the discussion that our model identifies patients who, despite receiving standard glucocorticoid therapy, remain at high risk of deterioration. This has direct clinical utility for risk stratification and the consideration of alternative or adjunctive immunomodulatory strategies, even if the outcome is a composite of both disease severity and drug response.

This study has some limitations. First, the findings are based on data from a single institution (First Hospital of Jilin University) and may reflect local treatment protocols or selection bias inherent to retrospective analyses. Second, the cohort size (*n* = 151) limits statistical power to detect rare events or subgroup-specific effects (e.g., diabetic subpopulations). Additionally, excluding 28 patients due to incomplete data may introduce selection bias. Third, Ferritin and IL-10 levels were measured only at admission. Dynamic changes during treatment, which may correlate with therapeutic response, were not analysed. Most importantly, our model was developed and validated on a relatively small sample from a single institution. The findings and the predictive performance of the nomogram may be influenced by local patient demographics and treatment protocols, and are therefore not immediately generalizable. External validation is a mandatory next step. Therefore, we expect the above limitations to be improved, and we are looking forward to using animal models or single-cell sequencing to clarify how IL-10 and ferritin modulate steroid resistance. Comparing high-dose (e.g., methylprednisolone >125 mg/day) versus standard regimens in hyperferritinemic patients is also important.

## 5 Conclusion

Overall, we identified independent risk factors for mortality in patients with COVID-19 treated with glucocorticoids and used these prognostic factors—diabetes, ferritin, and IL-10—to develop a nomogram model. This predictive model demonstrated favorable discrimination and calibration in our internal validation, highlighting its potential for early risk stratification to identify patients who remain at high risk for clinical deterioration despite standard glucocorticoid intervention. However, further validation in external, prospective cohorts is essential before it can be considered for broad clinical use.

## Data Availability

The data analyzed in this study is subject to the following licenses/restrictions: The data for our study were provided by Dan Li and are subject to licensing and authorization restrictions from the Department of Respiratory Medicine, the First Hospital of Jilin University. Therefore, they are not publicly available. However, we can consider requests for access on a case-by-case basis, contingent upon approval from the aforementioned department. Requests to access these datasets should be directed to the corresponding author.
