# Supplementary material for: Development of a laboratory-based nomogram for predicting clinical outcomes in patients with severe COVID-19 undergoing glucocorticoid therapy
Source: Front Med (Lausanne). 2025 Dec 5;12:1635545. doi: 10.3389/fmed.2025.1635545 (PMC12714636; doi:10.3389/fmed.2025.1635545)
Supplement: Supplementary file 1 [file Data_Sheet_1.pdf]

Supplementary Material

Table S1. Variance Inflation Factor (VIF) Values for Predictor Variables

Normal Range (VIF <10)

Variable	VIF Normal Range (VIF <10)	Variable	VIF Normal Range (VIF <10)
YEAR	2.02	DBP	2.79
Temperature (°C)	1.46	LY	6.84
Respiratory rate	1.56	MO	2.68
Pulse	1.7	HB	1.72
SBP	2.51	PLT	2.11
AST	5.11	PCT	1.9
ALT	5.08	D-D	45.54
γ-GT	1.82	FDP	45.83
ALB	1.14	APTT	1.72
GLB	1.78	PT	18.5
A/G	1.1	INR	16.97
TBA	2.6	IL-2	2.42
BUN	6.5	IL-4	2.86
CR	5.5	IL-6	1.47
Pro-BNP	2.34	IL-10	3.27

FET	2.37	TNF-α	1.38
CRP	2.79	Interferon-γ	1.26
IL-17A	1.94		

Extreme VIF (Excluded)

WBC	127.57
NE	108.4
TBIL	2.41×10^{6}
DBIL	5.89×10^{5}
IBLI	7.57×10^{5}

Notes:

Threshold: VIF <10 indicates acceptable collinearity (common threshold).

Excluded variables: WBC, NE, and bilirubin-related measures (TBIL, DBIL, IBLI) showed extreme VIFs (>100), likely due to formulaic relationships or measurement redundancy.

Coagulation markers (DD, FDP, PT, INR) exhibited elevated VIFs (16.97 – 45.83) but were retained based on clinical relevance.

Table S2. Diagnostic Performance Metrics of the Prediction Model

Metric	Estimate (95% CI)	Clinical Interpretation
Sensitivity	71.4% (59.4 – 81.6%)	Ability to correctly identify treatment failure cases (true positive rate)
Specificity	70.0% (60.0 – 78.8%)	Ability to correctly exclude non-failure cases (true negative rate)
PPV	62.5% (51.0 – 73.1%)	Proportion of predicted failures that are true failures
NPV	77.8% (67.8 – 85.9%)	Proportion of predicted non-failures that are true non-failures
Youden’s Index	0.414 (0.193 – 0.604)	Balanced measure of sensitivity and specificity (range: 0 – 1, higher = better)
Positive Likelihood Ratio (LR+)	2.38 (1.70 – 3.33)	Moderately increases probability of failure when test is positive

Negative Likelihood Ratio (LR−)	0.41 (0.28–0.60)	Significantly reduces probability of failure when test is negative
Diagnostic Odds Ratio (DOR)	5.83 (2.98 – 11.42)	Moderate overall discriminative ability (values >5 indicate clinical utility)

Diagnostic performance metrics of the prediction model for glucocorticoid therapy failure in COVID-19 patients (*n* = 151). Sensitivity, specificity, PPV, NPV, and Youden's index were calculated at the optimal probability threshold (0.5). Likelihood ratios and DOR quantify the model's ability to rule in/out treatment failure. Confidence intervals were derived using exact methods (Clopper-Pearson) and bootstrapping.

Formatting Notes: Units: (1) Sensitivity, specificity, PPV, NPV: **%** with one decimal place. (2) Likelihood ratios, DOR: 2 decimal places (except when <1.0, shown as 0.41). (3) Youden's Index: 3 decimal places (range 0–1). Confidence Intervals: (1) Calculated using the Clopper-Pearson exact method for proportions and bootstrapping (1,000 replicates) for LR/DOR. (2) Separated by en-dash (–) without spaces. Abbreviations: PPV: Positive Predictive Value, NPV: Negative Predictive Value, LR+: Positive Likelihood Ratio, LR−: Negative Likelihood Ratio, DOR: Diagnostic Odds Ratio.

Table S3. Description of Variables with Missing Data and Imputation Methods

Variable	Type	Missingness (%)	Imputation Method in mice
Total Bile Acid	Continuous	1%	Predictive Mean Matching
PCT	Continuous	5.9%	Predictive Mean Matching

Pro-BNP	Continuous	7.3%	Predictive Mean Matching
FET	Continuous	8%	Predictive Mean Matching
D-D	Continuous	4.6%	Predictive Mean Matching
FDP	Continuous	4.6%	Predictive Mean Matching
APTT	Continuous	1.9%	Predictive Mean Matching
PT	Continuous	1.9%	Predictive Mean Matching
INR	Continuous	1.9%	Predictive Mean Matching
Interferon γ	Continuous	1.3%	Predictive Mean Matching
IL-17A	Continuous	1.3%	Predictive Mean Matching

Table S4. Predictors for the nomogram in the Complete case

Intercept and variable	β	Odds ratios (95% CI)	P-value
Intercept	-1.7973		0.0032
IL-10	0.1189	2.13280 (1.0065, 4.5195)	0.0481
FET	0.001	2.30370 (1.0281, 5.1620)	0.0426
DM	0.9585	1.89 (1.29, 3.26)	0.0848
C-index			

Training cohort	0.78 (0.67–0.89)

Table S5. Comparison of Baseline Characteristics Between Patients Stratified by Serum Ferritin Level

Variables	FET＞970.7ug/L (n = 73)	FET＜970.7ug/L (n = 78)	p
Result, n (%)			< 0.001
No	21 (29)	57 (73)	
Yes	52 (71)	21 (27)	
SEX, n (%)			0.008
Female	21 (29)	40 (51)	
Male	52 (71)	38 (49)	
Hypertension, n (%)			0.459
No	33 (45)	41 (53)	
Yes	40 (55)	37 (47)	
Diabetes, n (%)			0.18
No	48 (66)	60 (77)	
Yes	25 (34)	18 (23)	

Cardiovascular disease, n (%)			0.961
No	54 (74)	59 (76)	
Yes	19 (26)	19 (24)	
Cerebral infarction, n (%)			0.237
No	68 (93)	67 (86)	
Yes	5 (7)	11 (14)	
Fever, n (%)			1
No	12 (16)	12 (15)	
Yes	61 (84)	66 (85)	
Cough, n (%)			0.69
No	12 (16)	10 (13)	
Yes	61 (84)	68 (87)	
Expectoration, n (%)			0.689
No	13 (18)	11 (14)	
Yes	60 (82)	67 (86)	
Dyspnea, n (%)			0.422
No	19 (26)	26 (33)	
Yes	54 (74)	52 (67)	
Chest Pain, n (%)			0.483

No	72 (99)	78 (100)	
Yes	1 (1)	0 (0)	
Hemoptysis, n (%)			0.682
No	71 (97)	74 (95)	
Yes	2 (3)	4 (5)	
Age (Year)	72 (67, 81)	71.5 (65, 78.75)	0.295
Temperature (° C)	38 (37.2, 38.5)	38.2 (37.02, 38.9)	0.305
Respiratory rate	20 (20, 22)	20 (20, 22)	0.629
Pulse	86 (79, 104)	88 (78.5, 99.75)	0.949
SBP (mmHg)	135.68 ±18.66	134.97 ± 22.23	0.831
DBP (mmHg)	78 (70, 85)	78 (70, 85)	0.884
WBC (109/L)	9 (5.37, 12.15)	8.6 (5.63, 11.23)	0.565
NE (109/L)	8.26 (4.37, 10.99)	7.21 (4.92, 9.65)	0.378
LY (109/L)	0.45 (0.29, 0.68)	0.56 (0.4, 0.83)	0.016
MO (109/L)	0.4 (0.24, 0.56)	0.39 (0.24, 0.68)	0.505
HB(g/L)	132 (121, 146)	126 (118.25, 137.75)	0.092
PLT (109/L)	169 (129, 248)	207.5 (154, 279)	0.023
AST (U/L)	37 (24.1, 59.3)	29.1 (22.02, 41.85)	0.042
ALT (U/L)	28.5 (16.4, 47.3)	28.7 (19.9, 42.6)	0.62

rGT (U/L)	75.5 (43, 177.8)	48.25 (29.18, 85.92)	0.011
ALB (g/L)	29.9 (27.4, 32.5)	31.7 (30.13, 34.65)	0.002
GLB (g/L)	28.71 卤 5.94	28.74 卤 4.36	0.969
AG	1.08 (0.91, 1.24)	1.12 (0.98, 1.25)	0.268
TBIL (umol/L)	14.3 (10.6, 18.1)	11.65 (9.05, 14.95)	0.003
DBIL (umol/L)	3.8 (2.6, 6.1)	2.6 (1.83, 4.07)	< 0.001
IBLI (umol/L)	10 (7.6, 12.8)	8.8 (7, 10.8)	0.03
TBA (umol/L)	5.2 (2.5, 7.1)	2.9 (1.92, 4.45)	0.002
BUN (mmol/L)	6.61 (4.95, 9.77)	5.98 (5.08, 7.79)	0.182
CR (umol/L)	66.6 (52.8, 93.5)	58.6 (46.9, 75.27)	0.029
CRP (mg/L)	109.34 (52.28, 185.49)	66.76 (23.06, 109.1)	0.001
PCT (ng/L)	0.23 (0.07, 1.55)	0.05 (0.05, 0.16)	< 0.001
PROBNP (pg/mL)	741 (321, 1690)	445 (136, 907)	0.004
DD (mg/L FEU)	1.43 (0.81, 3.59)	0.98 (0.45, 1.54)	< 0.001
FDP (ug/mL)	4.82 (3.39, 11.17)	2.9 (2.5, 5.47)	< 0.001
APTT (s)	29.2 (26.5, 31)	28.15 (25.25, 29.7)	0.029
PT (s)	12.4 (11.8, 13.3)	11.7 (11.12, 12.38)	< 0.001
INR	1.06 (0.99, 1.16)	1.01 (0.93, 1.07)	0.002
IL2 (pg/mL)	2.63 (1.99, 3.88)	2.29 (1.9, 2.97)	0.092

IL4 (pg/mL)	2.69 (1.99, 4.44)	2.35 (1.94, 3.03)	0.055
IL6 (pg/mL)	70.27 (31.46, 238.27)	31.71 (10.89, 94.05)	< 0.001
IL10 (pg/mL)	6.83 (4.8, 13.84)	3.94 (2.51, 7.47)	< 0.001
TNFa (pg/mL)	2.41 (1.92, 2.86)	2.18 (1.61, 2.69)	0.045
Interferon- γ (pg/mL)	3.14 (2.27, 5.05)	2.7 (2.06, 3.96)	0.067
IL17A (pg/mL)	7.17 (2.94, 14.16)	5.27 (2.8, 11.67)	0.253
